# Neurological manifestations and complications of coronavirus disease 2019 (COVID-19): a systematic review and meta-analysis

**DOI:** 10.1186/s12883-021-02161-4

**Published:** 2021-03-30

**Authors:** Ahmed Yassin, Mohammed Nawaiseh, Ala Shaban, Khalid Alsherbini, Khalid El-Salem, Ola Soudah, Mohammad Abu-Rub

**Affiliations:** 1 Division of Neurology, Department of Neurosciences, Faculty of Medicine, Jordan University of Science and Technology, P.O.Box 630001, Irbid, 22110 Jordan; 2 Graduate of The University of Jordan, Amman, Jordan; 3 Intern at Jordanian Royal Medical Services, Amman, Jordan; 4 Researcher, King Hussein Cancer Center, Amman, Jordan; 5 Department of Neurology, University of Tennessee Health Science Center, Methodist University Hospital, Memphis, TN USA; 6 Department of Basic Medical Sciences, Faculty of Medicine, Yarmouk University, Irbid, Jordan; 7 Department of Neurology, The George Washington University, Washington, DC USA

**Keywords:** COVID-19, Coronavirus, Neurology, CNS, Clinical features, Meta-analysis, Systematic review

## Abstract

**Background:**

The spectrum of neurological involvement in COVID-19 is not thoroughly understood. To the best of our knowledge, no systematic review with meta-analysis and a sub-group comparison between severe and non-severe cases has been published. The aim of this study is to assess the frequency of neurological manifestations and complications, identify the neurodiagnostic findings, and compare these aspects between severe and non-severe COVID-19 cases.

**Methods:**

A systematic search of PubMed, Scopus, EBSCO, Web of Science, and Google Scholar databases was conducted for studies published between the 1st of January 2020 and 22nd of April 2020. In addition, we scanned the bibliography of included studies to identify other potentially eligible studies. The criteria for eligibility included studies published in English language (or translated to English), those involving patients with COVID-19 of all age groups, and reporting neurological findings. Data were extracted from eligible studies. Meta-analyses were conducted using comprehensive meta-analysis software. Random-effects model was used to calculate the pooled percentages and means with their 95% confidence intervals (CIs). Sensitivity analysis was performed to assess the effect of individual studies on the summary estimate. A subgroup analysis was conducted according to severity. The main outcomes of the study were to identify the frequency and nature of neurological manifestations and complications, and the neuro-diagnostic findings in COVID-19 patients.

**Results:**

44 articles were included with a pooled sample size of 13,480 patients. The mean age was 50.3 years and 53% were males. The most common neurological manifestations were: Myalgia (22.2, 95% CI, 17.2 to 28.1%), taste impairment (19.6, 95% CI, 3.8 to 60.1%), smell impairment (18.3, 95% CI, 15.4 to 76.2%), headache (12.1, 95% CI, 9.1 to 15.8%), dizziness (11.3, 95% CI, 8.5 to 15.0%), and encephalopathy (9.4, 95% CI, 2.8 to 26.6%). Nearly 2.5% (95% CI, 1 to 6.1%) of patients had acute cerebrovascular diseases (CVD). Myalgia, elevated CK and LDH, and acute CVD were significantly more common in severe cases. Moreover, 20 case reports were assessed qualitatively, and their data presented separately.

**Conclusions:**

Neurological involvement is common in COVID-19 patients. Early recognition and vigilance of such involvement might impact their overall outcomes.

**Supplementary Information:**

The online version contains supplementary material available at 10.1186/s12883-021-02161-4.

## Background

Severe acute respiratory syndrome coronavirus 2 (SARS-CoV-2) has spread rapidly over the past year causing the Coronavirus Disease 2019 (COVID-19) pandemic. According to Johns Hopkins Coronavirus Resource Center, as of March 3, 2020, 192 nations and more than 114 million people across the globe have been affected while more than 2.5 million people died [1].

Although SARS-CoV-2 primarily affects the respiratory system causing pneumonia, multiorgan dysfunction and failure are likely to occur in severe cases [2]. There is mounting evidence that coronaviruses can invade the nervous tissue [3, 4] resulting in various neurological manifestations (NM) and neurological complications (NC) [5].

The literature about the NM of COVID-19 has been evolving with exponential increase in the number of publications. Multiple studies and case reports described the NM, which vary from being non-specific ones like headache, dizziness, and myalgias to more significant one like ataxia, seizures, anosmia, and ageusia [6–9]. Other studies reported NC of COVID-19 like acute ischemic stroke, cerebral venous sinus thrombosis, cerebral hemorrhage, and rhabdomyolysis [6, 10]. Abnormal findings in neurodiagnostic studies (ND) including neuroimaging (CT and MRI), cerebrospinal fluid (CSF) analysis, and neurophysiological studies (Electroencephalogram (EEG), Nerve Conduction Study (NCS), and Electromyography (EMG)) have also been described [6, 11, 12].

We conducted a systematic review and meta-analysis of studies addressing the neurological aspects of COVID-19 including NM, NC, and ND findings. In addition, we compared these aspects between severe and non-severe cases. Since the literature is still evolving and not many well designed studies have been published, we also performed a qualitative assessment of the case reports describing some unique NC of COVID-19.

## Methods

We developed a review protocol (registration number: PROSPERO CRD42020181298) prior to commencing the study. The Preferred Reporting Items for Systematic Reviews and Meta-Analyses (PRISMA) were used to ensure the reporting quality of this review [13].

### Literature search strategy

A broad search strategy was conducted through the following databases: PubMed, Scopus, EBSCO, Web of Science, and Google Scholar using terms related to COVID-19 and terms related to neurology; more details about the terms used in the search process are available in the appendix **(**Additional file 1**)**. Primary search process and secondary search process before the final analysis included studies published between January 1st 2020 and April 22nd 2020. Moreover, additional studies referenced in selected papers were identified and included.

### Inclusion and exclusion criteria

- Inclusion criteria:
  - Randomized controlled trials, non-randomized controlled trials, case-control studies, cohort studies, cross sectional studies, case series, and case reports.
  - Studies involving patients diagnosed with COVID-19, regardless of age.
  - Studies including clinical features of COVID-19 including NM, NC, or ND studies.
  - Articles published in English or are otherwise translated to English.
- Exclusion criteria:
  - Articles not addressing the neurological aspects of the infection.
  - Articles on cases with known neurological conditions before COVID-19 with no major neurological change during the infection (new symptoms or worsening of previous condition).
  - Studies addressing any of the other five human coronaviruses.
  - Studies published before 2020.

### Study selection

Four reviewers screened the titles and abstracts of retrieved records for eligibility using Rayyan software [14]. Individual studies were critically appraised by applying a standardized appraisal form appropriate for the study type. Inter-rater disagreements were resolved following a discussion between the reviewers.

### Data extraction

Two reviewers extracted the following information: date of publication, country, study design, age, gender, previous comorbidities, general and neurological clinical features, laboratory findings, imaging findings, neurophysiological study findings, severity and outcome of the disease. We tried to obtain unpublished missing data by contacting authors.

### Risk of Bias assessment

Two reviewers assessed the risk of bias using the NIH Study Quality Assessment Tools for case series, cross sectional and cohort studies [15, 16]. Conflicts were resolved by consulting a third reviewer.

### Data synthesis and analysis

We used a random effects model to calculate the pooled percentages for categorical variables and pooled means for continuous variables with their 95% confidence intervals (CIs) as the effect sizes. For data with median and inter-quartile range (IQR) or median and range, mean and standard deviation (SD) were calculated according to the equations by Luo et.al, Wan et.al, and Hozo et.al [17–19]. I^2^ statistic, T^2^ (tau-squared) test, and Cochrane Q were used to assess heterogeneity among studies. Data analysis was done using comprehensive meta-analysis software.

We assessed the existence of publication bias by the Egger’s test [20]. The existence of publication bias was determined by the degree of the funnel plot symmetry and we considered *P* < .05 as an evidence of the existence of publication bias.

### Subgroup and sensitivity analysis

A subgroup analysis was done to compare clinical and diagnostic neurological features in patients with severe disease compared to patients with non-severe disease; this categorization was determined if the study classified them into these groups Moreover, we performed a sensitivity analysis, in which the pooled estimates for each variable was recalculated, omitting one study at a time, to ensure that none of the included studies affected the results and to examine whether the overall effect size is statistically robust.

### Outcome measures

The main outcomes of this study were the frequency of NM, NC and ND findings. The main NM included but were not limited to: Headache, myalgia, weakness, dizziness, taste impairment (ageusia), smell impairment (anosmia), altered level of consciousness, behavioral changes, facial weakness, ataxia, abnormal movements (like tremor), hemiparesis, hemiplegia, vision impairment, cranial nerve dysfunction, numbness, paresthesia, and neuropathic pain. The NC included: Ischemic and hemorrhagic strokes, venous sinus thrombosis, meningitis, encephalitis, seizures, and rhabdomyolysis. The ND findings included: Laboratory findings (serum creatine kinase (CK), serum lactate dehydrogenase (LDH), neutrophil count, lymphocyte count, and monocyte count), CSF analysis, neuroimaging (MRI and CT), EEG, NCS, or EMG. Moreover, we examined the treatment associated neurological side effects or complications.

### Ratings of the quality of the evidence

According to the modified rating scale of Oxford Centre for Evidence-based Medicine for ratings of individual studies [21], the evidence for most of the studies in our meta-analysis was rated as level four (case series without intervention, and cross sectional) and only two were rated as level three (retrospective cohort studies). Moreover, we included case reports in our qualitative assessment (evidence level four; case reports).

## Results

### Study selection results

**Fig. 1:**
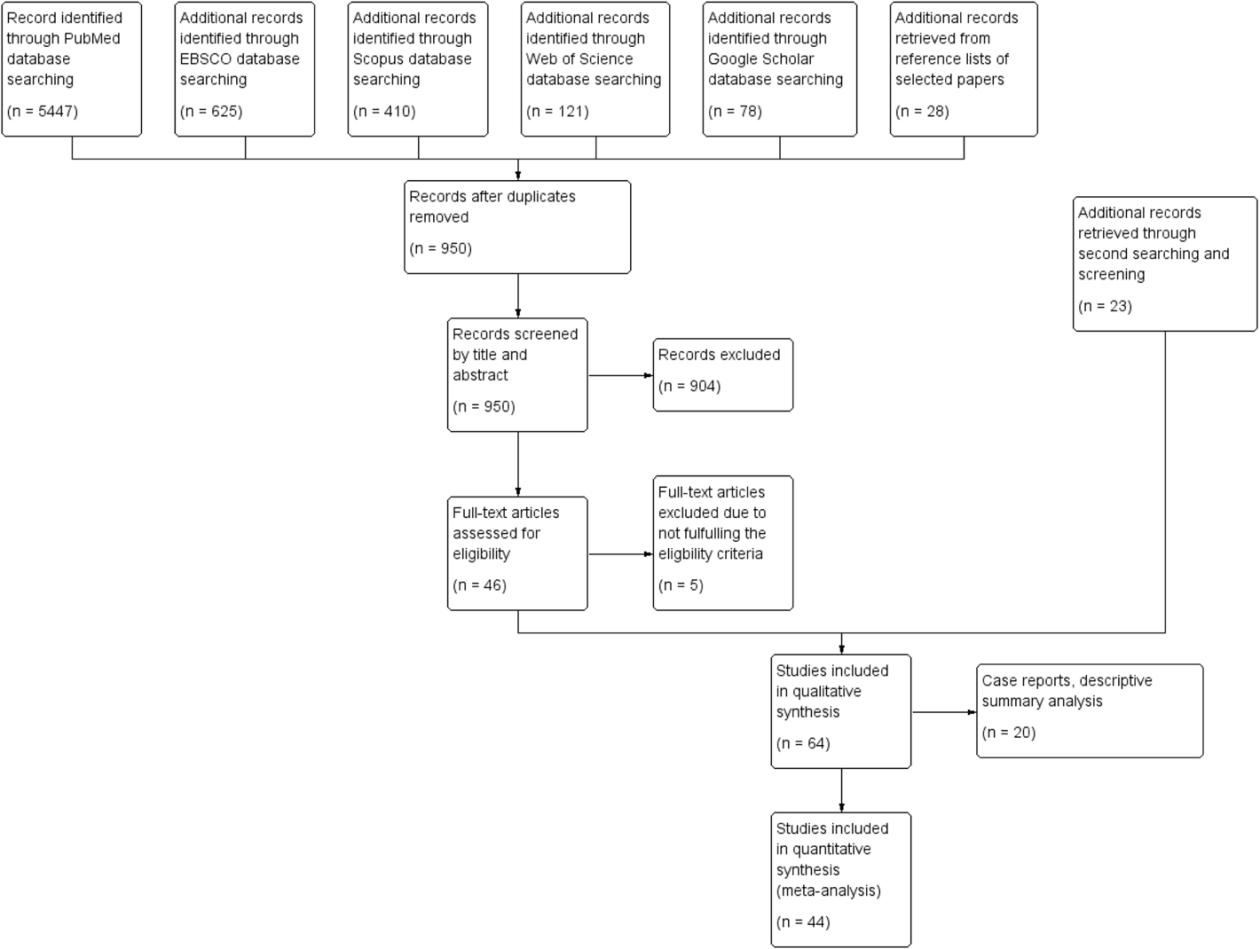
Flow diagram of study selection. Primary and secondary search processes yielded a total of 64 eligible articles. Forty-four articles were included in the final meta-analysis and 20 case reports were included in the qualitative descriptive review

The primary search yielded 6709 articles, with 41 articles remaining after removal of duplicates and screening titles, abstracts, and full texts. As a result of the rapid growth of the COVID-19 literature, a second search was conducted yielding another 23 articles. Forty-four articles were included in the final meta-analysis and 20 case reports were included in the qualitative descriptive review **(**Fig. 1**)**. Seventeen articles were available on the search databases but they were not yet published in their final form.

### Demographics and characteristics

Forty-four studies were included in the meta-analysis, 14 of which were available as pre-prints at the time of the search **(**Table 1**)**. A total of 13,480 patients were included in our analysis with a mean age of 50.3 (95% CI, 47.7 to 52.9) years, and 53% (95% CI, 50.2 to 55.7%) being males. Thirty-six (81.8%) studies were from China, two (4.5%) were from Italy, and the rest being one from each of Australia, France, Japan, Netherlands, Belgium and the UK. The study sample size ranged from 13 to 6606 patients per study.

**Table 1:** Characteristics of the Included Studies in the Meta-Analysis of the Neurological Features of COVID-19

#	Author	Date (DD/MM/Y)	Journal	Study type	N	Country	Reference	Study quality
1	Chen and Wu, 2020	27-3-2020	The Journal of Clinical Investigation	Case series	21	China	[22]	Fair
2	Liu and Zhang, 2020	Pre-print: 13-2-2020	The Lancet Infectious Diseases	Case series	24	China	[23]	Fair
3	Wang and Gao, 2020	Pre-proof: 5-3-2020	European Respiratory Journal	Case series	18	China	[24]	Fair
4	Giacomelli, 2020	26-3-2020	Clinical Infectious Diseases	Cross-Sectional Study	59	Italy	[25]	Fair
5	Mao, 2020	10-4-2020	JAMA Neurology	Case series	214	China	[6]	Fair
6	Xu and Yu, 2020	28-2-2020	European Journal of Nuclear Medicine and Molecular Imaging	Case series	90	China	[26]	Fair
7	Jin, 2020	24-3-2020	BMJ	Case series	651	China	[27]	Fair
8	Chen and Zhou, 2020	15-2-2020	The Lancet	Case series	99	China	[28]	Fair
9	Li and Li, 2020	Pre-print:12-2-2020	MDrxiv	Case series	17	China	[29]	Fair
10	Qian, 2020	17-3-2020	QJM	Case series	91	China	[30]	Fair
11	Xu and Wu, 2020	10-2-2020	BMJ	Case series	62	China	[31]	Fair
12	Huang and Wang, 2020	24-1-2020	Lancet	Case series	41	China	[32]	Fair
13	Wan, 2020	21-3-2020	Journal of Medical Virology	Case Series	135	China	[33]	Fair
14	Yang and Yu, 2020	24-2-2020	The Lancet Respiratory Medicine	Cohort - Retrospective	52	China	[34]	Fair
15	Liu and Fang, 2020	7-2-2020	Chinese Medical Journal	Case series	137	China	[35]	Fair
16	Guan, 2020	28-2-2020	The new england journal of medicine	Case series	1099	China	[2]	Fair
17	Wang and Hu, 2020	7-2-2020	JAMA	Case series	138	China	[36]	Fair
18	Qin and Qiu, 2020	Pre-print: 20-2-2020	TheLancet	Case series	89	China	[37]	Good
19	Yang and Cao, 2020	26-2-2020	The Journal of Infection	Case series	149	China	[38]	Fair
20	Qin and Zhou, 2020	12-3-2020	Clinical Infectious Diseases	Case series	452	China	[39]	Fair
21	Liu and Liu, 2020	12-2-2020	Preprint: medRxiv	Case series	61	China	[40]	Fair
22	Easom, 2020	29-3-2020	Influenza Other Respir Viruses	Case series	68	UK	[41]	Fair
23	Deng, 2020	20-3-2020	Chinese Medical Journal	Case series	225	China	[42]	Good
24	Huang and Tu, 2020	27-2-2020	Travel Medicine and Infectious Disease	Case series	34	China	[43]	Fair
25	Mo, 2020	16-3-2020	Clinical Infectious Diseases	Case series	155	China	[44]	Fair
26	Li and Wang, 2020	Pre-print:17-3-2020	The Lancet	Case series	221	China	[10]	Good
27	Zheng and Tang, 2020	24-3-2020	European Review for Medical and Pharmacological Sciences	Case series	161	China	[45]	Fair
28	Guo, 2020	Pre-print: 14-4-2020	The Lancet	Case series	118	China	[46]	Good
29	Yan, 2020	Pre-print: 6-4-2020	The Lancet	Case series	218	China	[47]	Good
30	Chang, 2020	17-3-2020	JAMA	Case series	13	China	[48]	Fair
31	Wang and Pan, 2020	Pre-proof: 11-4-2020	International Journal of Infectious Diseases	Case series	125	China	[49]	Fair
32	Zhou and Sun, 2020	Pre-print: 16-3-2020	BMC Infectious Diseases	Case series	201	China	[50]	Fair
33	Zheng and Xu, 2020	10-4-2020	Journal of Clinical Virology	Case series	99	China	[51]	Fair
34	Helms, 2020	15-4-2020	NEJM	Case series	58	France	[52]	Fair
35	Lechien, 2020	6-4-2020	European Archives of Oto-Rhino-Laryngology	Cross-Sectional Study	417	Belgium, France, Spain, Italy	[53]	Fair
36	Chen and Chen, 2020	Pre-print: 1-4-2020	The Lancet	Case series	85	China	[54]	Fair
37	Jiang, 2020	Pre-print: 14-4-2020	medRxiv	Case series	55	China	[55]	Good
38	Zhang, 2020	Pre-proof: 9-4-2020	Journal of Clinical Virology	Case series	221	China	[56]	Fair
39	Tabata, 2020	Pre-print: 18-3-2020	The Lancet	Case series	104	Japan	[57]	Fair
40	Lei, 2020	Pre-proof: 9-4-2020	Travel Medicine and Infectious Disease	Case series	20	Guangzhou, China	[58]	Fair
41	Zhou and Yu, 2020	28-3-2020	The Lancet	Cohort - Retrospective	191	China	[59]	Fair
42	Spinato, 2020	22-4-2020	JAMA	Cross-sectional Study	202	Italy	[60]	Fair
43	Klok, 2020	10-4-2020	Thrombosis Research	Case series	184	Netherlands	[61]	Fair
44	CNIRST, 2020	19-4-2020	NA	Case series	6606	Australia	[62]	Fair

*DD/MM/Y* Day, Month, Year. *NA* not applicable

The remaining 20 studies were included for the qualitative assessment of case reports **(**Table 2**)**, three of them were available as pre-prints at the time of the search. These case reports included 57 patients with a mean age of 59.5 (± 20.2) years and 38 (67%) being males.

**Table 2:** Characteristics of Included Case Reports

#	Author	Date (DD/MM/Y)	Journal	Study type	N	Country	Reference
1	Moriguchi, 2020	Pre-Print: 25-3-2020	International Journal of Infectious Diseases	Case Report	1	Japan	[11]
2	Zhao and huang, 2020	Pre-Print: 9-4-2020	medRxiv preprint	Case Report	1	China	[63]
3	Lorenzo Villalba, 2020	3-4-2020	European Journal of Case Reports in Internal Medicine	Case Report	2	France and Spain	[64]
4	Ollarves-Carrero, 2020	13-4-2020	Travel Medicine and Infectious Disease	Case Report	1	Spain	[65]
5	Sharifi-Razavi, 2020	27-3-2020	New Microbes and New Infections	Case Report	1	Iran	[66]
6	Marchese-Ragona, 2020	Pre-print: 7-4-2020	MedRxiv preprint	Case Report	6	Italy	[9]
7	Novi, 2020	9-4-2020	Multiple sclerosis and related disorders	Case Report	1	Italy	[67]
8	Poyiadji, 2020	31-3-2020	Radiology	Case Report	1	USA	[12]
9	Karimi, 2020	24-3-2020	Iran Red Crescent Med J	Case Report	1	Iran	[68]
10	Zhao and shen, 2020	1-4-2020	Lancet Neurology	Case Report	1	China	[69]
11	Gane, 2020	29-3-2020	Rhinology	Case Report	1	United Kingdom	[70]
12	Hjelmesæth, 2020	5-4-2020	Tidsskr Nor Legeforen	Case Report	3	Norway	[71]
13	Toscano, 2020	17-4-2020	NEJM	Case Report	5	Italy	[72]
14	Filatov, 2020	21-3-2020	Cureus	Case Report	1	USA	[8]
15	Suwanwongse, 2020	6-4-2020	Cureus	Case Report	1	USA	[73]
16	Wang and Hajizadeh, 2020	08-04-2020	Journal of Thrombosis and Haemostasis	Case Report	3	USA	[74]
17	Wang and Chen, 2020	09-02-2020	Bioscience Trends	Case Report	4	China	[75]
18	Ren, 2020	05-05-2020	Chinese Medical Journal	Case Report	5	China	[76]
19	Rothe, 2020	05-03-2020	NEJM	Case Report	1	Germany	[77]
20	Wang and Tang, 2020	27-01-2020	Journal of Medical Virology	Case Report	17	China	[78]

*DD/MM/Y* Day, Month, Year

### Risk of Bias assessment results

Of the 44 studies included in the meta-analysis, 39 were considered as case series and they were assessed for risk of bias using the NIH Quality Assessment Tool for Case Series Studies [16]. The study quality was rated as good, fair, or poor if the number of “Yes” responses were ≥ 6, 3 to 5, or ≤ 2, respectively. Of the 39-case series, 33 received a “fair” rating and 6 studies received a “good” rating.

Two studies were considered cohort studies and three were considered cross-sectional studies. They were assessed using the NIH Quality Assessment Tool for Observational Cohort and Cross-Sectional Studies [15]. The study quality was rated as good, fair, or poor if the number of “Yes” responses were ≥ 9, 4 to 8, or ≤ 3, respectively. All of the five included cohort and cross-sectional studies were given a “fair” rating.

Moreover, some questions of the previous quality assessment tools were not applicable to all studies. A more detailed illustration of the risk of bias assessment for each study is attached as a table in the supplementary appendix **(**Additional files 2 and 3**).**

### Clinical features and laboratory findings

The frequency of NM in COVID-19 patients was as follows: Myalgia (22.2, 95% CI, 17.2 to 28.1%), taste impairment (19.6, 95% CI, 3.8 to 60.1%), smell impairment (18.3, 95% CI, 15.4 to 76.2%), headache (12.1, 95% CI, 9.1 to 15.8%), dizziness (11.3, 95% CI, 8.5 to 15.0%), encephalopathy or cognitive dysfunction (9.4, 95% CI, 2.8 to 26.6%), and ataxia or abnormal gait (2.1, 95% CI, 0.2 to 23.7%). Nearly, 2.5% (95% CI, 1 to 6.1%) of COVID-19 patients had acute cerebrovascular diseases (CVD); which included ischemic stroke (IS), intracerebral hemorrhage (ICH), and cerebral venous sinus thrombosis (CVT) **(**Table 3**,** additional file 4**).**

**Table 3:** Meta-analysis of the clinical characteristics of the study subjects

	Pooled effect size (95% CI)	Heterogeneity			Tau squared	# of studies
		Q value	***P*** value	I Squared		
**Mean age (Years)**	50.3 (47.7–52.9)	2872.2	< .001	98.50	72.58	44
**Male**	53.0 (50.2–55.7) %	180.71	< .001	77.31	8.97	42
**Clinical features**						
**Headache**	12.1 (9.1–15.8) %	989.99	< .001	96.26	0.824	38
**Myalgia**	22.2 (17.2–28.1) %	621.55	< .001	94.85	0.740	33
**Taste impairment**	19.6 (3.8–60.1) %	431.04	< .001	99.30	3.405	4
**Smell impairment**	18.3 (1.54–76.2) %	853.88	< .001	99.64	7.254	4
**Dizziness**	11.3 (8.5–15.0) %	27.85	.001	67.68	0.156	10
**Features of encephalopathy or cognitive dysfunction**	9.4 (2.8–26.6) %	133.92	< .001	95.51	2.70	7
**Ataxia or abnormal gait**	2.1 (0.2–23.7) %	6.59	.010	84.83	3.18	2
**Fever**	80.6 (74.9–85.3) %	1604.55	< .001	97.44	1.05	42
**Cough**	64.1 (59.9–68.0) %	575.30	< .001	93.04	0.26	41
**Neurological complications** ^a^	3.0 (0.9–9.6) %	50.01	< .001	92.00	1.66	5
**Acute CVD**	2.5 (1.0–6.1) %	15.3	0.004	74.41	0.72	5
**Laboratory findings**						
**Serum CK (U/L)**	85.5 (73.8–97.3)	369.93	< .001	96.21	434.78	15
**Serum LDH (U/L)**	263.4 (234.6–292.3)	648.50	< .001	97.84	3026.56	15
**Lymphocyte (**^a^**10^9/L)**	1.08 (1.02–1.14)	549.37	< .001	95.08	0.024	28
**Neutrophils (**^a^**10^9/L)**	3.44 (3.21–3.68)	214.45	< .001	90.67	0.244	21
**Monocytes (**^a^**10^9/L)**	0.39 (0.37–0.42)	42.66	< .001	78.90	0.001	10
**Severe COVID-19**	31.1 (21.9–42.2) %	739.23	< .001	97.02	1.16	23
**ICU admission**	20.6 (14.1–29.0) %	231.12	< .001	91.34	0.81	21
**Comorbidities**						
**Any previous comorbidity**	37.4 (33.1–41.9) %	274.90	< .001	89.08	0.231	31
**Diabetes Mellitus**	10.3 (8.3–12.8) %	265.15	< .001	88.68	0.360	31
**Hypertension**	20.4 (17.0–24.2) %	196.73	< .001	87.292	0.253	26
**Heart diseases**	9.7 (7.2–12.9) %	426.59	< .001	93.201	0.706	30
**Neurological diseases**	5.7 (3.3–9.7) %	175.60	< .001	90.319	1.213	18
**Malignancy**	2.7 (2.0–3.6) %	61.429	< .001	59.303	0.319	26
**Pulmonary diseases**	3.4 (2.2–5.0) %	260.24	< .001	89.240	0.973	29
**Chronic kidney disease**	2.3 (1.3–3.9) %	75.189	< .001	81.380	0.858	15
**Chronic liver disease**	3.5 (2.6–4.7) %	32.726	.005	54.165	0.187	16
**Smoking**	9.2 (6.4–13.0) %	146.643	< .001	89.771	0.501	16

^a^Neurological complications include: Cerebrovascular diseases (ischemic stroke, cerebral hemorrhage, and venous sinus thrombosis), rhabdomyolysis, and seizures

P < .05 indicates the presence of heterogeneity

About a third of COVID-19 patients were severely affected (31.1, 95% CI, 21.9 to 42.2%) and 20.6% (95% CI, 14.1 to 29.0%) were admitted to intensive care units. About 37.4% (95% CI, 33.1 to 41.9%) had a pre-existing comorbidity, and 5.7% (95% CI, 3.3 to 9.7%) had a preexisting neurological disease. Detailed characteristics of the pre-existing comorbidities are presented in **(**Table 3**,** additional file 5**)**.

Regarding laboratory abnormalities **(**Table 3**,** additional file 6**)**, the mean values were as follows: CK: 85.57 U/L (Normal range; 40–200 U/L), LDH: 263.49 U/L (Normal range; 120–250 U/L). The mean lymphocyte, neutro0phil, and monocyte count were 1.08, 3.44, and 0.39 (*10^9/L), respectively.

No published data regarding COVID-19 treatment related neurological side effects and complications were found.

### Publication Bias

According to Egger et.al [20], publication bias assessment is only reliable for 10 or more pooled studies. Therefore, we presented the results of publication bias for variables that were discussed in 10 or more studies **(**Additional file 7**)**. Publication bias was observed in the following variables: fever (*p* < .001), headache (*p* < .001), serum LDH (*p* = .0015), Diabetes Mellitus (DM) (*p* = .0089), pre-existing neurological diseases (*p* = .0089), malignancy (*p* = .031), and chronic kidney disease (CKD) (*p* = .044).

### Sensitivity analysis

A sensitivity analysis, in which the meta-analysis was serially repeated after the exclusion of each study, demonstrated that no individual study affected the overall prevalence for each variable except for the following: Taste impairment prevalence was reduced from 19.6 to 10.9% when the study by Spinato et.al was excluded [60]; smell impairment prevalence was reduced from 18.3 to 7.5% when the study by Lechien et.al was excluded [53], and increased to 35.2% when the study by Mao et.al was removed [6]. After excluding the study conducted by Guan et.al, the reported frequency of NC increased from 3 to 5.8% [2]. More details can be found in additional file 8.

### Subgroup analysis

When comparing severe to non-severe COVID-19 patients, the severe group included older patients [mean age 60 vs 44.7 years-old, *p* < .001] and more males [60.3% vs 48.6%, *p* = .001] than the non-severe group. Myalgia [34.9% vs 4.1%, *p* = .045], acute CVD [34.9% vs 4.1%, *p* = .045], higher CK value [324.9 vs 121.2 U/L, *p* = .01], and higher LDH value (247.6 vs 83.0 U/L, *p* = .012) were more likely in the severe group. While encephalopathy and cognitive dysfunction were more frequent in the severe group [16.9% vs 1.9%, *p* = .054], this was not statistically significant. There was no significant difference for the rest of the variables evaluated **(**Table 4**)**. Heterogeneity was significant for all the variables and was not resolved by subgroup analysis.

**Table 4:** Subgroup analysis between severe and non-severe groups

Study	Subgroup	Pooled effect size (95% CI)	Heterogeneity				Tau squared	Mixed effects analysis
			**Q value**	**Df (Q)**	***P*** **value †**	**I Squared**		***P*** **value**
**Age (Years)**	Total	56.9 (55.1–58.8)	1443.18	34	< .001	97.64	107.603	< .001
	Non severe	44.4 (40.1–48.7)	585.98	16	< .001	97.26	77.40	
	Severe	60.0 (57.9–62.1)	78.77	17	< .001	78.418	13.35	
**Male**	Total	53.1 (49.5–56.6) %	108.58	31	< .001	71.45	0.104	.001
	Non severe	48.6 (44.2–53.1) %	54.23	15	< .001	72.34	0.082	
	Severe	60.3 (54.7–65.7) %	36.90	15	.001	59.36	0.104	
**Clinical features**								
**Headache**	Total	14.8 (12.4–17.5) %	187.25	30	< .001	83.97	0.474	.308
	Non severe	12.2 (7.9–18.2) %	170.26	15	< .001	91.19	0.730	
	Severe	15.4 (12.7–18.5) %	16.27	14	.296	14.003	0.025	
**Myalgia**	Total	24.4 (18.2–32.0) %	167.89	18	< .001	89.279	0.468	.045
	Non severe	19.4 (13.1–27.9) %	102.34	9	< .001	91.206	0.463	
	Severe	34.9 (22.3–49.9) %	58.061	8	< .001	86.221	0.651	
**Dizziness**	Total	11.9 (8.7–16.0) %	16.073	7	0.024	56.449	0.106	.506
	Non severe	10.9 (7.4–16.1) %	10.27	4	0.036	61.076	0.145	
	Severe	13.5 (8.2–21.5) %	5.619	2	0.06	64.409	0.152	
**Features of Encephalopathy / cognitive dysfunction**	Total	3.2 (1.2–8.4) %	116.97	6	< .001	94.87	4.753	.054
	Non severe	1.9 (0.6–5.8) %	2.266	2	.322	11.743	0.167	
	Severe	16.9 (2.4–62.3) %	83.34	3	< .001	96.4	4.342	
**Fever**	Total	79.8 (71.6–86.2) %	560.33	31	< .001	94.46	1.159	.213
	Non severe	76.9 (66.3–85.0) %	313.83	15	< .001	95.22	0.912	
	Severe	86.5 (72.6–93.9) %	238.40	15	< .001	93.708	2.63	
**Cough**	Total	59.2 (52.8–65.3) %	285.48	30	< .001	89.49	0.402	.094
	Non severe	55.8 (48.2–63.2) %	141.37	15	< .001	89.39	0.302	
	Severe	67.4 (55.9–77.2) %	135.46	14	< .001	89.66	0.734	
**Neurological Complications**	Total	3.8 (1.3–10.0) %	82.532	7	< .001	91.518	2.274	.212
	Non severe	1.3 (0.2–8.8) %	17.178	2	< .001	88.35	2.663	
	Severe	5.6 (1.7–17.1) %	37.55	4	< .001	89.34	1.607	
**Acute CVD**^a^	Total	2.6 (1.1–5.8) %	33.02	7	< .001	78.91	1.42	.045
	Non severe	0.6 (0.1–3.1) %	4.578	2	0.101	56.319	1.299	
	Severe	4.1 (1.6–10.0) %	15.38	4	0.004	74.00	0.797	
**Laboratory findings**								
**Serum CK**	Total	91.5 (79.3–103.7)	90.95	15	< .001	83.505	377.38	.01
	Non severe	83.0 (69.1–96.8)	53.346	7	< .001	86.87	276.03	
	Severe	121.2 (95.4–147.1)	18.80	7	< .001	62.76	633.03	
**Serum LDH**	Total	270.6 (243.1–298.1)	494.931	15	< .001	96.969	3099.14	.012
	Non severe	247.6 (214.8–280.4)	272.42	7	< .001	97.43	1997.9	
	Severe	324.9 (274.4–375.4)	66.42	7	< .001	89.462	4195.36	
**Preexisting neurological diseases**	Total	4.5 (2.8–7.0) %	101.58	20	< .001	80.31	1.055	.072
	Non severe	2.6 (1.2–5.5) %	36.692	9	< .001	78.19	0.970	
	Severe	6.2 (3.5–10.9) %	42.959	11	< .001	74.39	0.772	

^a^CVD (Cerebrovascular diseases): Ischemic stroke, cerebral hemorrhage, and venous sinus thrombosis

**†** P < .05 indicates the presence of heterogeneity

### Qualitative assessment

Twenty case reports (57 patients) were identified and their details are summarized in Table 5. Six (10.5%) patients were diagnosed with GBS 5–10 days after the onset of respiratory symptoms [69, 72]. Their neurological symptoms included numbness, weakness, dysphagia, and facial weakness; four patients (7.0%) had facial weakness including one (1.8%) with facial diplegia. All of these patients had abnormal NCS/EMG findings consistent with an axonal variant in three patients and a demyelinating variant in two.

**Table 5:** Patients characteristics and findings of the included case reports

Variable		N (%) or Mean ± SD	Variable		N (%) or Mean ± SD
Number	Cases	57	Clinical features	Fever	41 (71.9%)
	Articles	20		Cough	34 (59.6%)
**Countries of the cases reported**	China	28 (49.1%)		Fatigue	14 (25.6%)
	Italy	12 (21.0%)		Myalgia	12 (21.0%)
	USA	6 (10.5%)		Headache	5 (8.8%)
	Norway	3 (5.3%)		Dizziness	2 (3.5%)
	Iran	2 (3.5%)		Taste impairment	11 (19.3%)
	Spain	2 (3.5%)		Smell impairment	13 (22.8%)
	France	1 (1.8%)		Encephalopathy features	5 (8.8%)
	Germany	1 (1.8%)		Weakness/ paralysis	7 (12.3%)
	Japan	1 (1.8%)		Altered reflexes	3 (5.3%)
	UK	1 (1.8%)		Altered sensation^c^	5 (8.8%)
**Age (Years)**		59.5 ± 20.2		Ataxia or abnormal gait	1 (1.8%)
**Gender**	Male	38 (66.6%)		Facial weakness	4 (7%)
	Female	19 (33.3%)		Neck pain/ rigidity	2 (3.5%)
**Comorbidities**	Any	24 (42.1%)	**Number of neurological manifestations**	None	20 (35.0%)
	DM	7 (12.3%)		1–2	27 (47.3%)
	Hypertension	13 (22.8%)		> 3	10 (17.5%)
	Cardiovascular diseases	9 (15.7%)	**Neurological complications**	Any	12 (21.0%)
	Neurological diseases	8 (14.0%)		GBS	6 (10.5%)
	Chronic liver diseases	3 (5.2%)		Encephalitis	2 (3.5%)
	Pulmonary diseases	5 (8.8%)		Seizure	2 (3.5%)
	Malignancy or cancer	1 (1.8%)		Cerebral Hemorrhage	1 (1.8%)
	Chronic kidney disease	4 (7%)		Myelitis	1 (1.8%)
**ICU**	Yes	16 out of 28 (57.1%)		Rhabdomyolysis	1 (1.8%)
	No	12 out of 28 (42.8%)		Onset (Days)^a^	7.25 ± 2.43
	Onset (Days) ^a^	7.7 ± 2.9	**Imaging**	CT/MRI changes	6 (10.5%)
**Ventilator**	Yes	11 out of 31 (35.4%)	**CSF**	Increased protein	5 (8.8%)
	No	20 out of 31 (64.5%)		SARS-CoV-2 RNA in CSF	1 (1.8%)
	Onset (Days) ^a^	7 ± 2.49	**EEG**	Temporal slowing and sharp waves	1 (1.8%)
**Severity of COVID-19**	Asymptomatic	3 (5.3%)	**Nerve conduction study/EMG**	Demyelinating or Axonal patterns	6 (10.5%)
	Non-severe	19 (33.3%)	**Neurology-related management**		12 (21%)
	Severe	30 (52.6%)	**Neurological outcome**	Morbidity/ disability	4 out of 16(25%)
**COVID-19 disease outcome**	Death	20 out of 45(44.4%)		Recovery/ Improvement	10 out of 16(62.5%)
	Discharged/ Recovery	18 out of 45(40%)		Still hospitalized	2 out of 16(12.5%)
	Still hospitalized	7 out of 45(15.5%)		Onset (Days)^a,b^	15.5 (2.5)

Some data are missing or not reported. All patients in the aforementioned case reports were confirmed to have COVID-19

*GBS* Guillain–Barré Syndrome

^a^ Onset in relation to the onset of COVID-19 symptoms

^b^Reported as median and IQR

^c^ Altered sensation included paresthesia, numbness, loss of pain, temperature, or tactile sensations of the lower limbs, upper limbs, or trunk

Besides the above-mentioned EMG/NCS abnormalities, ND findings included neuro-imaging, CSF, and EEG findings. Neuro-imaging utilized were head CT, brain MRI and spinal MRI. Six patients had significant neuroimaging findings, including two patients with cerebral hemorrhage [12, 66], one patient with encephalitis/ventriculitis [11], two GBS patients with enhancement of the caudal nerve roots [72], and one GBS patient with bilateral enhancement of facial nerves [72]. Besides, six (10.5%) patients had CSF changes; mainly increased protein in five [8, 69, 72], and only one with SARS-CoV-2 RNA detected in CSF using RT-PCR assay [11]. Lastly, one patient had EEG changes consisting of bilateral and focal slowing in the left temporal region with left temporal sharp waves [8].

Twelve patients received neurology-related management including IVIG in eight patients, and four who used one or more of the following therapies: ceftriaxone, vancomycin, acyclovir, ganciclovir, steroids, levetiracetam, phenytoin, plasma exchange, or vitamin B12.

Of note, some NM and ND findings were reported by a few studies, out of the 44 studies, and were insufficient to be included in the meta-analysis. These included manifestations like visual impairment [6], nerve pain [6], and diffuse corticospinal tract signs with enhanced tendon reflexes, ankle clonus, and bilateral extensor plantar reflexes [52]. CSF findings included positive oligoclonal bands with the same pattern in serum, elevated CSF IgG and CSF protein levels, and low albumin level [52]. Head CT findings included ischemic stroke, cerebral hemorrhage, and cerebral venous sinus thrombosis [6, 10]. Brain MRI findings included leptomeningeal enhancement, bilateral frontotemporal hypoperfusion, and acute and subacute ischemic strokes [52]. EEG findings included nonspecific changes and slowing consistent with encephalopathy [52].

## Discussion

A total of 13,480 COVID-19 patients were included in the meta-analysis. NM were frequent with around 20% of patients reporting myalgia, taste impairment, or smell impairment; and around 10% complaining of headache, dizziness, or encephalopathy. Ataxia or abnormal gait was the least reported NM. Five studies reported NC (CVD, seizures, and rhabdomyolysis). CVDs (IS, ICH, CVT) occurred in 2.5% of patients. For those who were tested, high levels of CK and LDH as markers of muscle injury were found, especially in the severe subgroup. About one third of patients included in this study had severe disease course and one fifth of them were admitted to the ICU.

There is a mounting evidence that Angiotensin Converting Enzyme 2 (ACE 2) receptors are expressed throughout the central nervous system, primarily on the surface of neurons [79], and SARS-CoV-2 might use these receptors to gain entry into the nervous system [3, 4, 80]. The result of direct neuronal invasion could explain manifestations such as headache, dizziness, ataxia and encephalopathy, while neuronal death and inflammation could explain complications like meningitis/encephalitis [11, 81], as well as seizures or even refractory status epilepticus [82–84]. Interestingly, direct invasion of the respiratory centers in the brainstem was proposed as a contributing factor to the respiratory failure in COVID-19 patients [3, 85].

Viral entry into the CNS is debatable. This could happen via a hematogenous route in which the virus passes through the blood brain barrier (BBB) by transcytosis or infects endothelial or epithelial cells to cross the BBB [4, 11, 86]. Alternatively, the virus could infect and get transported by leukocytes into the CNS, as was shown for SARS-CoV [87].

Moreover, ACE 2 receptor is heavily expressed on the epithelial cells of the mucosa of the oral cavity [88] and a trans-neural transmission of SARS-CoV through the olfactory bulb was seen in a mice model [89]. Sungnak et al. surveyed expression of SARS-CoV-2 viral entry-associated genes in multiple tissues from healthy human donors and found these genes highly expressed in nasal epithelial cells [90]. These findings could explain the occurrence of anosmia and ageusia in COVID-19 patients, which at times can be the only presenting features or the very early symptoms of COVID 19 [53, 91].

Myalgia and occasionally clinically significant muscle injury in severe disease, as evidenced by elevated CK and LDH, can be either a direct response of viral invasion of the skeletal muscles, which are also known to express ACE2 receptor [80], or an indirect response to the systemic inflammatory reaction manifested by a cytokine storm, subsequently causing muscle injury [92–94].

Multiple mechanisms could explain the increased risk of ischemic strokes and venous sinus thrombosis [95, 96]; these include hypercoagulability [6, 97], high systemic inflammatory response or “cytokine storm” [98], vascular endothelial injury [59], and cardiac injury resulting in cerebral embolism [99]. It is worth-mentioning there were anecdotal reports of decline in stroke admission rates in certain communities, possibly due to the anxiety surrounding this pandemic which discourages patients, especially those with mild stroke symptoms, from seeking emergency medical services [100–104]. There is a need for clear guidelines for the neuroradiology departments on how to safely and effectively perform urgent neuro-diagnostic images and emergent neuro-interventional procedures [100, 105, 106]. Implementing such guidelines are critical to streamline the management of COVID-19 patients presenting with neurological complications such as stroke, and to maintain a high-quality standard workflow.

According to our analysis, myalgia and evidence of muscle injury “elevated CK and LDH” as well as CVD were more likely to occur with severe disease. This might be related to the degree of the inflammatory response and the reported cytokine release syndrome [107] as well as the prothrombotic state [108] that occur with severe cases of COVID-19 and contribute to the multiorgan failure [22, 109].

Congruent with what Mao et al. [6] reported in the first retrospective observational case series describing the NM of COVID-19 in 214 hospitalized patients in Wuhan-China, our meta-analysis shows that myalgia or skeletal muscle injury (with elevated LDH and CK) and acute CVDs are predominantly associated with severe COVID-19.

A recent systematic review of 8 studies [110], not including a meta-analysis, suggested that some patients, particularly those with severe illness, have CNS involvement and NM, which is supported by the results of our study. Montalvan et al. [111] concluded that symptoms of hyposmia, headaches, weakness, and altered consciousness, and complications like encephalitis, demyelination, neuropathy, and stroke were associated with coronaviruses infections. Those results are congruent with our findings, although we looked at SARS-CoV-2 exclusively, while they evaluated other human coronaviruses in addition. The authors also suggested that trans-synaptic extension through the cribriform plate and olfactory bulb represents the main mechanism of neuro-invasion, and that invasion of the medulla could contribute to the respiratory failure in critically ill COVID-19 patients. A group from the National Hospital, Queen Square described five major categories of NM and NC associated with COVID-19, including: (i) encephalopathies with delirium/psychosis in the absence of characteristic MRI or CSF abnormalities; (ii) inflammatory CNS syndromes including encephalitis, acute disseminated encephalomyelitis which many times was hemorrhagic, and myelitis; (iii) ischemic strokes (half of them with pulmonary embolism); (iv) peripheral neuropathies including Guillain-Barré Syndrome (GBS) and brachial plexopathy; and (v) miscellaneous central nervous system disorders [112]. Ahmad et al. [113] in a narrative literature review reported that neurological features could occur before the classical features of COVID-19 like fever and cough, and accordingly a high index of suspicion is needed for a timely diagnosis and isolation of cases.

In the 20 case reports we evaluated, the most common NM included fatigue, myalgia, and smell and taste impairment, which is quite similar to our meta-analysis results. NC included GBS (6 cases), encephalitis, seizures, ICH, IS, myelitis and rhabdomyolysis. GBS associated with COVID-19 indicates that SARS CoV-2 can potentially induce an immune response that results in a delayed neurological complication [114]. This association between coronaviruses and GBS was reported before [114, 115]. In these case reports, the neurological outcome was variable, but one fourth of patients were left with residual deficits after 2 weeks of COVID-19 disease onset, indicating potential severity of the neurological injury.

### Quality of the evidence

We believe that the evidence generated from our meta-analysis is reliable since it is based on fair to good quality studies and well-defined search methods and eligibility criteria. More than 40 studies in varied populations have been included in the final meta-analysis, with emphasis on avoiding overlapping data. In addition, we performed a subgroup analysis to test if there is an association between neurological manifestations of COVID-19 and severity of the disease. We followed the Preferred Reporting Items for Systematic Reviews and Meta-Analyses (PRISMA) checklist to prepare this study [13].

### Limitations

Limitations of our analysis include the heterogeneity among the studies being considerably high both in the overall population and following the subgroup analysis. This is due to the large variation in the sample size among studies, the different study designs and methodologies, lack of uniformity in collecting and reporting of data, and possibly reflecting a true variation between different populations. Sensitivity analysis was conducted to explore the heterogeneity. Moreover, random effect model was set a priori since significant heterogeneity was expected. Besides, most of the included studies collected the data retrospectively. Finally, egger test indicated that there is a possible publication bias among the following variables: Fever, headache, serum LDH, DM, pre-existing neurological diseases, malignancy, and CKD. There is a possibility that some unpublished studies were not identified as our meta-analysis was limited to studies published in English-language and since many studies were not yet published at the time of screening. However, we tried to avoid publication bias by including studies translated into English as well as including pre-prints and contacting authors.

## Conclusion

In this meta-analysis on the neurological features of COVID-19, we found that several NM and NC are associated with COVID-19, and certain features, such as CVD, muscle injury, and probably encephalopathy, might be associated with severe disease status. Healthcare professional dealing with COVID-19, neurologists, and the general public should be aware of the neurological involvement of the disease. Patients of possible COVID-19 presenting with the previously mentioned neurological features should trigger clinical suspicion. Further studies are required to assess the prevalence of the neurological aspects of COVID-19 in different populations and to directly compare them between severe and non-severe subgroups. More pathophysiological analysis and studies are required as well in order to understand the exact mechanism through which the virus affects the nervous system.

## Supplementary Information

**Additional file 1.**

**Additional file 2.**

**Additional file 3.**

**Additional file 4.**

**Additional file 5.**

**Additional file 6.**

**Additional file 7.**

**Additional file 8.**

**Additional file 9.**

## Data Availability

All data synthesized and analyzed are included in this published article.

## Abbreviations

EEG: Electroencephalography
EMG: Electromyography
CK: Creatine Kinase
CNS: Central Nervous System
COVID-19: Coronavirus Disease 2019
CSF: Cerebrospinal Fluid
CT: Computed Tomography
LDH: Lactate Dehydrogenase
MRI: Magnetic Resonance Imaging
NCS: Nerve Conduction Study
SARS-CoV-2: Severe Acute Respiratory Syndrome Coronavirus 2
PRISMA: Preferred Reporting Items for Systematic Reviews and Meta-Analyses
BBB: Blood Brain Barrier
NM: Neurological Manifestations
NC: Neurological Complications
ND: Neurodiagnostic
