# Supplementary material for: Neurological manifestations and complications of coronavirus disease 2019 (COVID-19): a systematic review and meta-analysis
Source: BMC Neurol. 2021 Mar 30;21:138. doi: 10.1186/s12883-021-02161-4 (PMC8007661; doi:10.1186/s12883-021-02161-4)
Supplement: Supplementary file 1 — Additional file 1. [file 12883_2021_2161_MOESM1_ESM.docx]

**Methods 1: Detailed information about the terms used in the search process**

We used the following keywords in our search process: (COVID-19 or SARS-CoV-2 or COVID 2019 or Novel coronavirus or Novel coronavirus 2019 or 2019 nCoV or Wuhan coronavirus or Wuhan pneumonia) and (neurology or neurological or neurological manifestations or neurological features or epidemiological or clinical features or clinical characteristics or nervous or CNS or brain or cranial nerve or headache or encephalopathy or confusion or delirium or personality changes or aphasia or facial weakness or diplopia or dysarthria or dysphagia or dysphonia or hearing loss or vision loss or anosmia or ageusia or motor weakness or sensory deficit or myalgia or seizures or meningitis or encephalitis or stroke or cerebral hemorrhage or cerebral or venous thrombosis or rhabdomyolysis or CSF analysis or brain MRI or head CT or EEG or NCS or EMG).
