# Supplementary material for: Neurological manifestations and complications of coronavirus disease 2019 (COVID-19): a systematic review and meta-analysis
Source: BMC Neurol. 2021 Mar 30;21:138. doi: 10.1186/s12883-021-02161-4 (PMC8007661; doi:10.1186/s12883-021-02161-4)
Supplement: Supplementary file 2 — Additional file 2. [file 12883_2021_2161_MOESM2_ESM.docx]

**NIH Quality Assessment Tool for Case Series Studies**

| **Author** | **Q1** | **Q2** | **Q3** | **Q4** | **Q5** | **Q6** | **Q7** | **Q8** | **Q9** | **Quality rating** |
| --- | --- | --- | --- | --- | --- | --- | --- | --- | --- | --- |
| Chen and Wu, 2020 | Yes | Yes | Yes | N/A | N/A | No | No | Yes | Yes | Fair |
| Liu and Zhang, 2020 | Yes | Yes | Yes | N/A | N/A | No | CD | Yes | Yes | Fair |
| Wang and Gao, 2020 | No | Yes | Yes | N/A | N/A | No | No | No | Yes | Fair |
| Mao, 2020 | Yes | Yes | Yes | N/A | N/A | No | No | Yes | Yes | Fair |
| Xu and Yu, 2020 | Yes | Yes | Yes | N/A | N/A | No | No | Yes | Yes | Fair |
| Huang and Wang, 2020 | Yes | Yes | Yes | N/A | N/A | No | No | Yes | Yes | Fair |
| Jin, 2020 | Yes | Yes | Yes | N/A | N/A | No | CD | Yes | Yes | Fair |
| Chen and Zhou, 2020 | Yes | Yes | Yes | N/A | N/A | No | No | Yes | Yes | Fair |
| Li and Li, 2020 | Yes | Yes | Yes | N/A | N/A | No | CD | Yes | Yes | Fair |
| Qian, 2020 | Yes | Yes | Yes | N/A | N/A | No | No | Yes | Yes | Fair |
| Xu and Wu, 2020 | Yes | Yes | Yes | N/A | N/A | No | No | No | Yes | Fair |
| Wan, 2020 | Yes | Yes | Yes | N/A | N/A | No | No | Yes | Yes | Fair |
| Liu and Fang, 2020 | Yes | Yes | Yes | N/A | N/A | No | No | Yes | Yes | Fair |
| Guan, 2020 | Yes | Yes | Yes | N/A | N/A | No | No | Yes | Yes | Fair |
| Wang and Hu, 2020 | Yes | Yes | Yes | N/A | N/A | No | No | Yes | Yes | Fair |
| Qin and Qiu, 2020 | Yes | Yes | Yes | N/A | N/A | No | Yes | Yes | Yes | Good |
| Yang and Cao, 2020 | Yes | Yes | Yes | N/A | N/A | No | No | Yes | Yes | Fair |
| Qin and Zhou, 2020 | Yes | Yes | Yes | N/A | N/A | No | CD | Yes | Yes | Fair |
| Liu and Liu, 2020 | Yes | Yes | Yes | N/A | N/A | No | No | Yes | Yes | Fair |
| Easom, 2020 | Yes | Yes | Yes | N/A | N/A | No | CD | No | Yes | Fair |
| Deng, 2020 | Yes | Yes | Yes | N/A | N/A | No | Yes | Yes | Yes | Good |
| Huang and Tu, 2020 | Yes | Yes | Yes | N/A | N/A | No | CD | No | Yes | Fair |
| Mo, 2020 | Yes | Yes | Yes | N/A | N/A | No | CD | Yes | Yes | Fair |
| Li and Wang, 2020 | Yes | Yes | Yes | N/A | N/A | Yes | No | Yes | Yes | Good |
| Zheng and Tang, 2020 | Yes | Yes | Yes | N/A | N/A | No | No | Yes | Yes | Fair |
| Cheng, 2020 | Yes | Yes | Yes | N/A | N/A | No | Yes | Yes | Yes | Good |
| Yan, 2020 | Yes | Yes | Yes | N/A | N/A | No | Yes | Yes | Yes | Good |
| Chang, 2020 | Yes | Yes | Yes | N/A | N/A | No | Yes | No | Yes | Fair |
| Wang and Pan, 2020 | Yes | Yes | Yes | N/A | N/A | No | No | Yes | Yes | Fair |
| Zhou and Sun, 2020 | Yes | Yes | Yes | N/A | N/A | No | No | Yes | Yes | Fair |
| Zheng and Xu, 2020 | Yes | Yes | Yes | N/A | N/A | No | CD | Yes | Yes | Fair |
| Helms, 2020 | Yes | Yes | Yes | N/A | N/A | No | CD | No | Yes | Fair |
| Chen and Chen, 2020 | Yes | Yes | Yes | N/A | N/A | No | No | No | Yes | Fair |
| Jiang, 2020 | Yes | Yes | Yes | N/A | N/A | No | Yes | Yes | Yes | Good |
| Zhang, 2020 | Yes | Yes | Yes | N/A | N/A | No | No | Yes | Yes | Fair |
| Tabata, 2020 | Yes | Yes | Yes | N/A | N/A | No | No | Yes | Yes | Fair |
| Lei, 2020 | Yes | Yes | Yes | N/A | N/A | No | CD | Yes | Yes | Fair |
| Klok, 2020 | Yes | Yes | Yes | N/A | N/A | Yes | No | No | Yes | Fair |
| CNIRST, 2020 | Yes | No | Yes | N/A | N/A | No | CD | No | Yes | Fair |

CD, cannot determine; NA, not applicable; NR, not reported.
**Q1**; Was the study question or objective clearly stated? / **Q2**; Was the study population clearly and fully described, including a case definition? / **Q3**; Were the cases consecutive?  / **Q4**; Were the subjects comparable?  / **Q5**; Was the intervention clearly described?  / **Q6**; Were the outcome measures clearly defined, valid, reliable, and implemented consistently across all study participants?  / **Q7**; Was the length of follow-up adequate?  / **Q8**; Were the statistical methods well-described?  / **Q9**; Were the results well-described?
