# Supplementary material for: Neurological manifestations and complications of coronavirus disease 2019 (COVID-19): a systematic review and meta-analysis
Source: BMC Neurol. 2021 Mar 30;21:138. doi: 10.1186/s12883-021-02161-4 (PMC8007661; doi:10.1186/s12883-021-02161-4)
Supplement: Supplementary file 4 — Additional file 4. [file 12883_2021_2161_MOESM4_ESM.docx]

**General and Neurological Manifestations and Complications in Patients Included in the Meta-Analysis.**

| **Author** | **N** | **General** | | **Neurological** | | | | | | | | | | | **Neurological complications** | |
| --- | --- | --- | --- | --- | --- | --- | --- | --- | --- | --- | --- | --- | --- | --- | --- | --- |
|  |  | **Fever** | **Cough** | **Number of neurological manifestations** | **Headache** | **Myalgia** | **Taste impairment** | **Smell impairment** | **Dizziness** | **Encephalopathy Features/ cognitive dysfunction** | **Ataxia or Abnormal Gait** | **Seizure** | **Vision impairment** | **Nerve pain** | **any** | **Acute CVD** |
| Chen and Wu, 2020 | 21 | 20 | 16 | 3 | 2 | 8 |  |  |  | 3 |  |  |  |  |  |  |
| Liu and Zhang, 2020 | 24 | 19 | 6 | 3 | 4 | 2 |  |  | 4 |  |  |  |  |  |  |  |
| Wang and Gao, 2020 | 18 | 17 | 10 | 2 | 1 | 1 |  |  |  |  |  |  |  |  |  |  |
| Giacomelli, 2020 | 59 | 43 | 22 | 4 | 2 |  | 17 | 14 |  |  |  |  |  |  |  |  |
| Mao, 2020 | 214 | 132 | 107 | 10 | 28 |  | 12 | 3 | 36 | 16 | 1 | 1 | 3 | 5 | 30 | 6 |
| Xu and Yu, 2020 | 90 | 70 | 57 | 2 | 4 | 25 |  |  |  |  |  |  |  |  |  |  |
| Jin, 2020 | 651 | 545 | 435 | 2 | 67 | 71 |  |  |  |  |  |  |  |  |  |  |
| Chen and Zhou, 2020 | 99 | 82 | 81 | 3 | 8 | 11 |  |  |  | 9 |  |  |  |  |  |  |
| Li and Li, 2020 | 17 | 12 | 13 | 2 |  | 4 |  |  | 2 |  |  |  |  |  |  |  |
| Qian, 2020 | 91 | 65 | 55 | 2 | 7 | 5 |  |  |  |  |  |  |  |  |  |  |
| Xu and Wu, 2020 | 62 | 48 | 50 | 2 | 21 | 32 |  |  |  |  |  |  |  |  |  |  |
| Huang and Wang, 2020 | 41 | 40 | 31 | 2 | 3 | 18 |  |  |  |  |  |  |  |  |  |  |
| Wan, 2020 | 135 | 120 | 102 | 2 | 34 | 44 |  |  |  | 14 |  |  |  |  |  |  |
| Yang and Yu, 2020 | 52 | 51 | 40 | 2 | 3 | 6 |  |  |  |  |  |  |  |  |  |  |
| Liu and Fang, 2020 | 137 | 112 | 66 | 2 | 13 | 44 |  |  |  |  |  |  |  |  |  |  |
| Guan, 2020 | 1099 | 975 | 745 | 2 | 150 | 164 |  |  |  |  |  |  |  |  | 2 | 0 |
| Wang and Hu, 2020 | 138 | 136 | 82 | 3 | 9 | 48 |  |  | 13 |  |  |  |  |  |  |  |
| Qin and Qiu, 2020 | 89 | 86 | 83 | 3 | 15 | 30 |  |  | 16 |  |  |  |  |  |  |  |
| Yang and Cao, 2020 | 149 | 114 | 87 | 2 | 13 | 5 |  |  |  |  |  |  |  |  |  |  |
| Qin and Zhou, 2020 | 452 | 423 | 152 | 4 | 52 | 98 |  |  | 37 | 3 |  |  |  |  |  |  |
| Liu and Liu, 2020 | 61 | 60 | 39 | 1 | 21 |  |  |  |  |  |  |  |  |  |  |  |
| Easom, 2020 | 68 | 27 | 53 | 2 | 3 | 11 |  |  |  |  |  |  |  |  |  |  |
| Deng, 2020 | 225 | 189 | 85 | 2 | 13 | 57 |  |  |  |  |  |  |  |  |  |  |
| Huang and Tu, 2020 | 34 | 32 | 17 | 2 | 2 | 22 |  |  |  |  |  |  |  |  |  |  |
| Mo, 2020 | 155 | 126 | 97 | 3 | 8 | 50 |  |  | 2 |  |  |  |  |  |  |  |
| Li and Wang, 2020 | 221 |  |  |  |  |  |  |  |  |  |  |  |  |  | 13 | 13 |
| Zheng and Tang, 2020 | 161 | 122 | 101 | 2 | 12 | 18 |  |  |  |  |  |  |  |  |  |  |
| Cheng, 2020 | 118 | 82 | 81 | 5 | 15 | 60 |  |  | 15 | 5 | 8 |  |  |  |  |  |
| Yan, 2020 | 218 | 145 | 162 | 2 | 28 | 41 |  |  |  |  |  |  |  |  |  |  |
| Chang, 2020 | 13 | 12 | 6 | 2 | 3 | 3 |  |  |  |  |  |  |  |  |  |  |
| Wang and Pan, 2020 | 125 | 116 | 102 | 3 | 11 | 4 |  |  | 11 |  |  |  |  |  |  |  |
| Zhou and Sun, 2020 | 201 | 135 | 118 | 2 | 18 | 21 |  |  |  |  |  |  |  |  |  |  |
| Zheng and Xu, 2020 | 99 | 85 | 84 | 2 | 12 | 12 |  |  |  |  |  |  |  |  |  |  |
| Helms, 2020 | 58 | 8 |  | 6 |  |  |  |  |  | 40 |  |  |  |  | 3 | 3 |
| Lechien, 2020 | 417 | 200 | 325 | 5 | 188 | 242 |  | 357 |  |  |  |  |  |  |  |  |
| Chen and Chen, 2020 | 85 | 55 | 37 | 1 | 4 |  |  |  |  |  |  |  |  |  |  |  |
| Jiang, 2020 | 55 | 47 | 34 | 1 | 10 |  |  |  |  |  |  |  |  |  |  |  |
| Zhang, 2020 | 221 | 200 | 136 | 1 | 17 |  |  |  |  |  |  |  |  |  |  |  |
| Tabata, 2020 | 104 | 36 | 43 | 1 | 18 |  |  |  |  |  |  |  |  |  |  |  |
| Lei, 2020 | 20 | 16 | 11 | 1 |  | 7 |  |  |  |  |  |  |  |  |  |  |
| Zhou and Yu, 2020 | 191 | 180 | 151 | 1 |  | 29 |  |  |  |  |  |  |  |  |  |  |
| Spinato, 2020 | 202 | 113 | 122 | 4 | 86 | 90 | 130 |  | 28 |  |  |  |  |  |  |  |
| Klok, 2020 | 184 |  |  | 1 |  |  |  |  |  |  |  |  |  |  | 3 | 3 |
| CNIRST, 2020 | 6,606 | 3170 | 4624 | 3 | 2378 |  | 494 | 526 |  |  |  |  |  |  |  |  |
| Total | 13480 | 8266 | 8668 |  | 3283 | 1283 | 653 | 900 | 164 | 90 | 9 | 1 | 3 | 5 | 51 | 25 |

CVD, cerebrovascular disease
