# Supplementary material for: Neurological manifestations and complications of coronavirus disease 2019 (COVID-19): a systematic review and meta-analysis
Source: BMC Neurol. 2021 Mar 30;21:138. doi: 10.1186/s12883-021-02161-4 (PMC8007661; doi:10.1186/s12883-021-02161-4)
Supplement: Supplementary file 5 — Additional file 5. [file 12883_2021_2161_MOESM5_ESM.docx]

**Demographic Data of the Patients Included and their Baseline Comorbidities.**

| **Author** | **N** | **Mean age (Years)** | **Male (N)** | **Comorbidities** | | | | | | | | | | **Severe** | **ICU admission** |
| --- | --- | --- | --- | --- | --- | --- | --- | --- | --- | --- | --- | --- | --- | --- | --- |
|  |  |  |  | **Any comorbidity** | **DM** | **HTN** | **Heart disease** | **Neurolog-ical diseases** | **Malignancy** | **Pulmonary diseases** | **CKD** | **Chronic liver disease** | **Smoking** |  |  |
| Chen and Wu, 2020 | 21 | 57 | 17 | 7 | 3 | 5 |  |  |  |  |  |  |  | 11 |  |
| Liu and Zhang, 2020 | 24 | 43 | 8 |  |  |  |  |  |  |  |  |  |  | 0 |  |
| Wang and Gao, 2020 | 18 | 41.16 | 10 | 6 | 3 | 5 | 3 | 2 | 1 |  |  |  |  |  |  |
| Giacomelli, 2020 | 59 | 61.41 | 40 |  |  |  |  |  |  |  |  |  |  |  |  |
| Mao, 2020 | 214 | 52.7 | 87 | 83 | 30 | 51 | 15 | 15 | 13 |  | 6 |  |  | 88 |  |
| Xu and Yu, 2020 | 90 | 50 | 39 | 45 | 5 | 17 | 3 |  | 2 | 3 |  |  |  |  |  |
| Jin, 2020 | 651 | 45.2 | 331 | 178 | 48 | 100 |  |  | 6 | 1 | 6 | 25 |  | 64 | 17 |
| Chen and Zhou, 2020 | 99 | 55.5 | 67 | 50 | 12 |  | 40 | 41 | 1 | 1 |  |  |  |  | 23 |
| Li and Li, 2020 | 17 | 45.1 | 9 | 3 |  | 1 |  |  |  |  |  |  | 3 |  |  |
| Qian, 2020 | 91 | 47.71 | 37 | 26 | 8 | 15 | 3 | 3 |  |  |  |  |  | 9 | 9 |
| Xu and Wu, 2020 | 62 | 41.7 | 35 | 20 | 1 | 5 |  | 1 |  | 1 | 1 | 7 |  |  | 1 |
| Huang and Wang, 2020 | 41 | 49.35 | 30 | 13 | 8 | 6 | 6 |  | 1 | 1 |  | 1 | 3 |  | 13 |
| Wan, 2020 | 135 | 45.94 | 72 | 43 | 12 | 13 | 7 |  | 4 | 1 |  | 2 | 9 | 40 |  |
| Yang and Yu, 2020 | 52 | 59.7 | 35 | 21 | 9 |  | 5 | 7 | 2 | 4 |  |  | 2 | 52 | 52 |
| Liu and Fang, 2020 | 137 | 57 | 61 | 27 | 14 | 13 | 10 |  | 2 | 2 |  |  |  |  |  |
| Guan, 2020 | 1099 | 46.64 | 637 | 261 | 81 | 165 | 27 |  | 10 | 12 | 8 | 23 | 137 | 173 |  |
| Wang and Hu, 2020 | 138 | 55.29 | 75 | 64 | 14 | 43 | 20 | 7 | 10 | 4 | 4 | 4 |  |  | 36 |
| Qin and Qiu, 2020 | 89 | 54.64 | 45 | 25 | 10 | 15 | 3 |  | 2 | 5 | 1 | 5 | 21 |  | 35 |
| Yang and Cao, 2020 | 149 | 45.11 | 81 | 52 |  |  | 28 | 28 | 2 | 1 |  |  |  |  | 0 |
| Qin and Zhou, 2020 | 452 | 57.29 | 235 | 201 | 75 | 135 | 27 | 11 | 14 | 12 | 10 | 6 | 7 | 286 |  |
| Liu and Liu, 2020 | 61 | 40 | 31 | 27 | 5 | 12 | 1 |  |  | 5 |  |  | 4 | 17 |  |
| Easom, 2020 | 68 | 42.5 | 36 |  |  |  |  |  |  |  |  |  |  |  |  |
| Deng, 2020 | 225 | 54.04 | 124 | 127 | 26 | 58 | 17 |  | 8 | 25 |  |  |  |  |  |
| Huang and Tu, 2020 | 34 | 56 | 14 | 16 | 4 | 8 | 6 |  |  | 3 |  | 1 |  |  | 8 |
| Mo, 2020 | 155 | 54 | 86 |  | 15 | 37 | 15 | 7 | 7 | 5 | 6 | 7 | 6 | 92 |  |
| Li and Wang, 2020 | 221 | 53.3 | 131 | 85 | 31 | 55 | 17 | 17 | 14 |  |  |  | 5 | 11 |  |
| Zheng and Tang, 2020 | 161 | 45.17 | 80 | 33 | 7 | 22 | 4 | 4 |  | 6 |  | 4 |  | 30 |  |
| Cheng, 2020 | 118 | 71.68 | 53 |  | 27 | 64 | 23 |  | 2 | 13 | 6 | 1 |  |  |  |
| Yan, 2020 | 218 | 42.37 | 122 |  | 27 |  | 38 | 6 | 2 | 14 | 4 | 13 | 23 | 38 | 25 |
| Chang, 2020 | 13 | 39.11 | 10 |  |  |  |  |  |  |  |  |  |  |  | 0 |
| Wang and Pan, 2020 | 125 | 38.76 | 71 | 34 |  |  | 18 | 1 | 1 | 2 |  |  | 16 | 25 | 19 |
| Zhou and Sun, 2020 | 201 | 45.98 | 102 |  |  |  | 41 |  |  | 41 |  |  |  |  | 45 |
| Zheng and Xu, 2020 | 99 | 49.4 | 51 | 41 | 6 |  | 21 |  |  | 1 |  | 5 |  | 32 | 32 |
| Helms, 2020 | 58 | 63 |  |  |  |  |  | 7 |  |  |  |  |  | 58 | 58 |
| Lechien, 2020 | 417 | 36.9 | 154 |  |  |  |  |  |  |  |  |  | 56 |  |  |
| Chen and Chen, 2020 | 85 | 40.11 | 34 |  | 3 | 6 |  | 1 | 1 | 1 | 1 |  |  | 9 | 7 |
| Jiang, 2020 | 55 | 43.93 | 27 | 29 | 9 | 17 | 2 | 1 | 2 |  | 1 | 2 |  | 8 |  |
| Zhang, 2020 | 221 | 53.42 | 108 | 78 | 22 | 54 | 22 | 15 | 9 | 6 | 6 | 7 |  | 55 | 44 |
| Tabata, 2020 | 104 | 62.98 | 47 | 52 | 7 |  | 31 |  | 4 | 7 |  |  | 18 | 28 |  |
| Lei, 2020 | 20 | 43.2 | 10 | 7 |  |  |  |  |  | 1 |  |  |  |  | 1 |
| Zhou and Yu, 2020 | 191 | 56.35 | 119 | 91 | 36 | 58 | 15 |  | 2 | 6 | 2 |  | 11 | 119 | 50 |
| Spinato, 2020 | 202 | 56 | 97 | 113 |  |  |  |  |  |  | 23 |  | 63 |  |  |
| Klok, 2020 | 184 | 64 | 139 |  |  |  |  |  | 5 |  |  |  |  | 184 | 184 |
| CNIRST, 2020 | 6,606 | 46.5 |  |  | 311 |  | 354 |  |  | 153 |  |  |  |  |  |
| Total | 13480 |  |  | 1858 | 869 | 980 | 822 | 174 | 127 | 337 | 85 | 113 | 384 | 1429 | 659 |

DM, Diabetes mellitus; HTN, Hypertension; CKD, Chronic kidney disease.
