# Supplementary material for: Neurological manifestations and complications of coronavirus disease 2019 (COVID-19): a systematic review and meta-analysis
Source: BMC Neurol. 2021 Mar 30;21:138. doi: 10.1186/s12883-021-02161-4 (PMC8007661; doi:10.1186/s12883-021-02161-4)
Supplement: Supplementary file 6 — Additional file 6. [file 12883_2021_2161_MOESM6_ESM.docx]

**Laboratory Findings of the Study Patients Included in the Meta-Analysis.**

| **Author** | **CK (Mean)** | **CK (SD)** | **LDH (Mean)** | **LDH (SD)** | **lymphocyte count (Mean)** | **lymphocyte count (SD)** | **Neutrophils count (Mean)** | **Neutrophils count (SD)** | **Monocytes count (Mean)** | **Monocytes count (SD)** |
| --- | --- | --- | --- | --- | --- | --- | --- | --- | --- | --- |
| Chen and Wu, 2020 | 146.2 | 178 | 366.8 | 251.2 | 0.9 | 0.318 | 4.8359 | 3.2596 |  |  |
| Liu and Zhang, 2020 |  |  |  |  |  |  |  |  |  |  |
| Wang and Gao, 2020 |  |  |  |  |  |  |  |  |  |  |
| Giacomelli, 2020 |  |  |  |  |  |  |  |  |  |  |
| Mao, 2020 | 467.5 | 2213.8 | 255.7 | 164.2 | 1.1 | 0.4167 | 3 | 3.1167 |  |  |
| Xu and Yu, 2020 |  |  |  |  |  |  |  |  |  |  |
| Jin, 2020 |  |  |  |  | 1.1 | 0.4 |  |  |  |  |
| Chen and Zhou, 2020 | 107.8 | 100 | 348.3 | 140.6 | 0.9 | 0.5 | 5.49 | 3.6111 |  |  |
| Li and Li, 2020 |  |  |  |  | 1.1 | 0.5 | 4.6 | 2.8 | 0.4 | 0.2 |
| Qian, 2020 |  |  |  |  | 1.32 | 0.512 | 2.9311 | 0.9642 | 0.4282 | 0.2109 |
| Xu and Wu, 2020 | 70.2 | 45.9 | 217.1 | 58 | 1.1 | 0.53 | 2.8647 | 1.2903 |  |  |
| Huang and Wang, 2020 | 138.1 | 120.6 | 313.6 | 127.5 | 0.835 | 0.384 | 5.7805 | 4.3022 |  |  |
| Wan, 2020 | 95.6 | 67.4 | 317.9 | 102.5 | 1.1 | 0.5995 | 3.5 | 1.3488 |  |  |
| Yang and Yu, 2020 |  |  |  |  | 0.68 | 0.3851 |  |  |  |  |
| Liu and Fang, 2020 |  |  |  |  |  |  |  |  |  |  |
| Guan, 2020 |  |  |  |  | 1 | 0.44 |  |  |  |  |
| Wang and Hu, 2020 | 92.7 | 55.4 | 283.1 | 165.5 | 0.835 | 0.3746 | 3.3163 | 2.1726 | 0.4 | 0.1498 |
| Qin and Qiu, 2020 |  |  |  |  | 0.8 | 0.4 | 3.5 | 1.1666 | 0.4 | 0.15 |
| Yang and Cao, 2020 | 76.5 | 53.5 | 210 | 94.5 | 1.21 | 0.68 | 2.6 | 2.03 |  |  |
| Qin and Zhou, 2020 |  |  |  |  | 0.9 | 0.4462 | 4.1103 | 2.3798 | 0.4 | 0.1487 |
| Liu and Liu, 2020 | 95.8 | 60.7 |  |  | 1.07 | 0.4556 | 2.7119 | 1.063 | 0.3 | 0.1519 |
| Easom, 2020 |  |  |  |  |  |  |  |  |  |  |
| Deng, 2020 |  |  |  |  |  |  |  |  |  |  |
| Huang and Tu, 2020 |  |  |  |  |  |  |  |  |  |  |
| Mo, 2020 | 99.3 | 34.4 | 292.8 | 156.3 | 0.88 | 0.336 | 3.1008 | 1.8259 |  |  |
| Li and Wang, 2020 |  |  |  |  |  |  |  |  |  |  |
| Zheng and Tang, 2020 | 63 | 20.5 | 180.1 | 60.5 | 1.091 | 0.493 |  |  |  |  |
| Cheng, 2020 |  |  |  |  |  |  |  |  |  |  |
| Yan, 2020 |  |  | 236.77 | 216.84 | 1.25 | 0.61 |  |  |  |  |
| Chang, 2020 |  |  |  |  | 1.58 | 0.653 | 3.67 | 1.71 | 0.5253 | 0.3472 |
| Wang and Pan, 2020 | 67.2 | 35.6 | 246.2 | 63.3 | 1.0382 | 0.4463 | 3.2858 | 1.6013 | 0.3716 | 0.1875 |
| Zhou and Sun, 2020 | 81.3 | 55.4 | 174.8 | 54.2 | 1.1702 | 0.5974 | 2.9 | 1.3441 |  |  |
| Zheng and Xu, 2020 |  |  |  |  | 1.81 | 1.26 | 4.31 | 2.46 |  |  |
| Helms, 2020 |  |  |  |  |  |  |  |  |  |  |
| Lechien, 2020 |  |  |  |  |  |  |  |  |  |  |
| Chen and Chen, 2020 |  |  |  |  | 1.9 | 0.7541 | 3.3761 | 1.5837 |  |  |
| Jiang, 2020 |  |  |  |  | 1.4095 | 0.7308 | 3.0135 | 1.0582 | 0.4835 | 0.1903 |
| Zhang, 2020 | 95.4 | 65.6 | 257.5 | 144.01 | 0.8351 | 0.3731 | 3.3509 | 2.3879 | 0.4 | 0.1492 |
| Tabata, 2020 |  |  |  |  |  |  |  |  |  |  |
| Lei, 2020 |  |  |  |  | 1.4 | 0.7 |  |  |  |  |
| Zhou and Yu, 2020 | 36.3 | 44.3 | 314.3 | 129.2 | 0.9649 | 0.5229 |  |  |  |  |
| Spinato, 2020 |  |  |  |  |  |  |  |  |  |  |
| Klok, 2020 |  |  |  |  |  |  |  |  |  |  |
| CNIRST, 2020 |  |  |  |  |  |  |  |  |  |  |

CK, Creatine kinase; LDH, Lactate Dehydrogenase.
