# Supplementary material for: Neurological manifestations and complications of coronavirus disease 2019 (COVID-19): a systematic review and meta-analysis
Source: BMC Neurol. 2021 Mar 30;21:138. doi: 10.1186/s12883-021-02161-4 (PMC8007661; doi:10.1186/s12883-021-02161-4)
Supplement: Supplementary file 7 — Additional file 7. [file 12883_2021_2161_MOESM7_ESM.docx]

**Egger's Test for Publication Bias.**

| **Variable** | **P value (2-tailed)** |
| --- | --- |
| Mean age (Years) | .346 |
| Male | .696 |
| Clinical features |  |
| Fever | < .001 |
| Cough | .177 |
| Headache | < .001 |
| Myalgia | .383 |
| Taste impairment | < 10 studies |
| Smell impairment | < 10 studies |
| Dizziness | .452 |
| Encephalopathy features or cognitive dysfunction | < 10 studies |
| Ataxia or abnormal gait | Two studies only |
| Neurological complications | < 10 studies |
| Acute CVD | < 10 studies |
| Severe | .934 |
| ICU admission | .893 |
| Comorbidities |  |
| Any previous comorbidity | .267 |
| DM | .0089 |
| HTN | .624 |
| Heart disease | .113 |
| Neurological Diseases | .0089 |
| Malignancy | .031 |
| Pulmonary diseases | .946 |
| CKD | .044 |
| Chronic liver disease | .768 |
| Smoking | .069 |
| Laboratory findings |  |
| Serum CK (U/L) | .069 |
| Serum LDH (U/L) | .00158 |
| Lymphocyte (* 10^9/L) | .111 |
| Neutrophils (* 10^9/L) | .056 |
| Monocytes (* 10^9/L) | .795 |

DM, Diabetes Mellitus; HTN, Hypertension; CKD, Chronic Kidney Disease; CVD, Cerebrovascular Disease; CK, Creatine Kinase; LDH, Lactate Dehydrogenase. P < .05 indicates the presence of publication bias.
