# Supplementary material for: Neurological manifestations and complications of coronavirus disease 2019 (COVID-19): a systematic review and meta-analysis
Source: BMC Neurol. 2021 Mar 30;21:138. doi: 10.1186/s12883-021-02161-4 (PMC8007661; doi:10.1186/s12883-021-02161-4)
Supplement: Supplementary file 8 — Additional file 8. [file 12883_2021_2161_MOESM8_ESM.docx]

**Results of the Sensitivity Analysis.**

| **Variable** | **Change** |
| --- | --- |
| Taste impairment | Removing Spinato et al study will make the prevalence 10.9 % (4.5% to 24.3%) |
| Smell impairment | Removing Lechien et al study will make the prevalence 7.5 % (2.5% to 20.7%)  Removing Mao et al study will make the prevalence 35.2 % (2.4% to 92.4%) |
| Neurological complications | Removing Guan et al study will make the prevalence 5.8 % (2.4% to 13.1%) |

There were no significant changes in the pooled effect size for all the variables upon conducting sensitivity analysis, except for the mentioned variables above.
